# Rectification and Super-Resolution Enhancements for Forensic Text Recognition †

**DOI:** 10.3390/s20205850

**Published:** 2020-10-16

**Authors:** Pablo Blanco-Medina, Eduardo Fidalgo, Enrique Alegre, Rocío Alaiz-Rodríguez, Francisco Jáñez-Martino, Alexandra Bonnici

**Affiliations:** 1 Department of Electrical, Systems and Automation, Universidad de León, 24007 León, Spain; eduardo.fidalgo@unileon.es (E.F.); enrique.alegre@unileon.es (E.A.); rocio.alaiz@unileon.es (R.A.-R.); fjanm@unileon.es (F.J.-M.); 2 INCIBE (Spanish National Cybersecurity Institute), 24005 León, Spain; 3 Faculty of Engineering, University of Malta, MSD2080 Msida, Malta; alexandra.bonnici@um.edu.mt

**Keywords:** text spotting, text recognition, super-resolution, Tor Darknet, computer forensics

## Abstract

Retrieving text embedded within images is a challenging task in real-world settings. Multiple problems such as low-resolution and the orientation of the text can hinder the extraction of information. These problems are common in environments such as Tor Darknet and Child Sexual Abuse images, where text extraction is crucial in the prevention of illegal activities. In this work, we evaluate eight text recognizers and, to increase the performance of text transcription, we combine these recognizers with rectification networks and super-resolution algorithms. We test our approach on four state-of-the-art and two custom datasets (TOICO-1K and Child Sexual Abuse (CSA)-text, based on text retrieved from Tor Darknet and Child Sexual Exploitation Material, respectively). We obtained a 0.3170 score of correctly recognized words in the TOICO-1K dataset when we combined Deep Convolutional Neural Networks (CNN) and rectification-based recognizers. For the CSA-text dataset, applying resolution enhancements achieved a final score of 0.6960. The highest performance increase was achieved on the ICDAR 2015 dataset, with an improvement of 4.83% when combining the MORAN recognizer and the Residual Dense resolution approach. We conclude that rectification outperforms super-resolution when applied separately, while their combination achieves the best average improvements in the chosen datasets.

## 1. Introduction

The automatic detection, segmentation and recognition of text in natural images, also known as text spotting, is a challenging task with multiple practical applications [1,2,3]. The location and transcription of text may be a great aid in forensic applications such as the analysis of Child Sexual Abuse Material (CSAM), the investigation of domains of the Tor network or the retrieval of critical information from criminal scenes among other tasks [4,5].

Although specialized analysts in forensic laboratories can easily recognize multiple objects and text in an image with little or no conscious effort, this manual analysis becomes unfeasible within the proposed time constraints of most investigations [6]. Manual text-spotting is not only a very time-consuming and expensive task, but it also exposes analysts to sensitive data on a daily basis, affecting their emotional state, therefore hindering their performance. Thus, the development and implementation of fast, automatic and efficient tools for the analysis of images and videos become crucial for the forensic field [4,5].

Artificial vision techniques [7] allow a high-level understanding of digital images or videos without the need for a human operator. Furthermore, the availability of huge amounts of data, hardware resources and machine learning techniques (both traditional and those based on deep learning) allows for the development of models that can extract meaningful information from images. Thus, multiple objects can be detected and classified within an image with high performance. Image classification techniques allow for the identification of specific content [8], which can be further explored through object detection [9,10,11] and object recognition techniques [12,13].

Text spotting helps enhance the task of analyzing multimedia material from sensitive environments, such as CSAM or The Onion Router (Tor) darknet. The concept of darknet refers to those networks from the Dark Web that require the use of specific browsers to be accessed. The Tor network is one such darknet inside of the dark web, offering user anonymity thanks to its layered domains, also known as hidden services. Due to this high level of privacy and anonymity, Tor is commonly used by journalists to protect their sources, IT professionals to test the security of their networks and other users wishing to remain anonymous.

**Figure 1:**
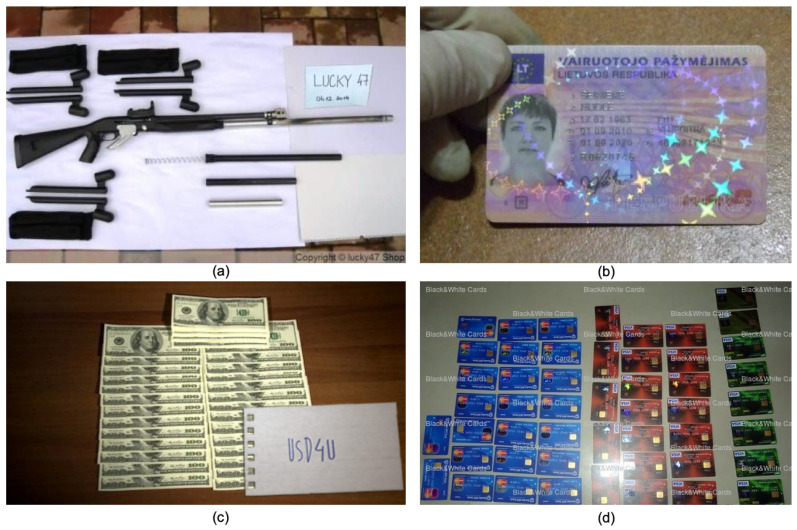
Images crawled from Tor darknet. Samples from (**a**) dismantled weapon, (**b**) fake id, (**c**) fake money and (**d**) credit cards.

However, Tor darknet also hosts suspicious content and services, such as traders selling different kinds of unregulated products. Al-Nabki et al. [14] reported that 29% of the active domains crawled from Tor darknet, during their study, contained different kinds of suspicious or potentially illegal activities. Figure 1 gives examples of content typically found on Tor darknet and which includes domains with weapons, drug selling, or personal identification counterfeiting.

Different research efforts have been made to monitor Tor domains, and insights gained have been used to develop techniques that allow for the supervision of suspicious activities. These techniques range from solutions based on Natural Language Processing [15,16], to Computer Vision [17,18] or Graph Theory [19,20].

However, some of the Computer Vision approaches overlook the analysis of the text found within the images, which could be an important source of information. In Tor, depending on the context or activity hosted in the hidden service, images could contain text that provides additional information such as drug names or seller names, among others, as shown in Figure 1. Likewise, CSAM text retrieval may provide details regarding the offender, which can be classified afterwards to acquire additional information relevant to law enforcement agencies [21].

To the best of our knowledge, there are only two works that focus on the application of text spotting to forensic applications. In the first, Blanco et al. [22] obtained an F-Measure of 0.57 in the text detection task, but their performance on text recognition with state-of-the-art techniques was much lower. Through the use of dictionaries and string-matching techniques, Blanco et al. [23] improved the score of the recognition stage to 0.3970.

**Figure 2:**
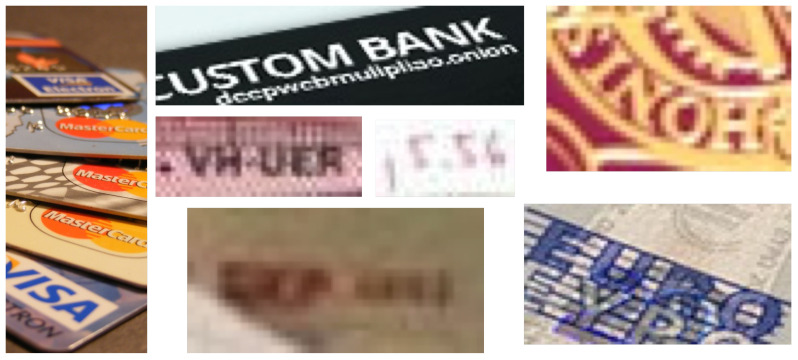
Common problems found in Tor images. Orientation (**left**, **middle-top** and **right**) and low-resolution (**middle**).

We can attribute the lower score obtained in these works [22,23] to different image quality factors, such as colour distribution, brightness, and partial occlusion, some of which we illustrate in Figure 2. Other problems affecting the text recognition task include multiple fonts or languages in the same image, character similarity, lighting conditions or even mistakes when labelling the images [24]. Although these are frequent problems in state-of-the-art datasets, the issues of low resolution and oriented text are the most remarkable in both CSA and Tor-based images [23]. In Tor images, it is very common to fit several documents in a single picture to express quantity or product variations. This problem becomes worse if, after the text is detected, the image is cropped to include only the text content. In such cases, the quality of the cropped section can decrease further depending on the file format chosen when saving the image. Such degradations in image quality reduce the performance of text recognizers.

In this paper, we address the problem of performing text recognition on non-horizontal and low-resolution text [25], by enhancing images using two different techniques; rectification networks [26,27], which correct an image’s orientation to reduce transcription mismatches, and super-resolution techniques, which improve the image quality before recognition. We combine these two tasks on CSA focused images as well as on Tor darknet images [22,23] in order to retrieve information that can be of use to identify potentially illegal activities.

We selected a total of eight text recognition algorithms, studying their performance on four state-of-the-art datasets and two custom datasets; namely CSA-text, a dataset that contains text retrieved from CSA-material, and TOICO-1K, a Tor-based image dataset we released and made publicly available. We perform text recognition on single-region images, obtained from the ground truth of each dataset. The two best performing approaches, which are based on rectification networks [26,27], are later combined with three state-of-the-art super-resolution techniques, increasing text recognition performance.

The rest of the paper is organized as follows. Section 2 presents related work on text recognition and text-based super-resolution approaches. Section 3 describes the selected recognizers, rectification and super-resolution based methods as well as the datasets used, while Section 4 details our experiments and compares the results of combining these techniques. Lastly, we present our conclusions and future lines of work for the further improvement of text recognition.

## 2. Related Work

Text spotting is the joint task of detecting areas inside an image or video that contain text, followed by their transcription into a legible character sequence. This task can be used in different applications, such as automatic navigation and document analysis [1]. After extraction, text can be further analyzed using Natural Language Processing (NLP) techniques [28].

Text retrieval can be hindered by several issues, including lighting problems, character similarity or curved text. Text can also appear inside images with low resolution, which can be hard to transcribe accurately. Researchers have studied the most relevant problems in both detection and recognition [1,2], establishing orientation and segmentation amongst the most common problems [24].

Currently, most approaches focus on the combination of Convolutional Neural Networks (CNN) with Recurrent Neural Networks (RNN). Shi et al. [29] proposed the first end-to-end recognition system based on this combination, creating a framework that combined sequence modelling, feature extraction and transcription. This proposal was able to handle sequences of arbitrary length on a smaller model while achieving good performance in both lexicon and non-lexicon based recognition.

The combination of both text detection and text recognition into a single system has attracted great interest from researchers. Liu et al. [30] proposed a unified trainable network that performs better than separate approaches due to sharing features between both stages using a RoiRotate operator, reducing computational costs. While these approaches often achieve good performance on most datasets, they have difficulties treating irregular text, most notably in the recognition stage.

There are two approaches to enhance the recognition step, namely the bottom-up and the top-down approaches [27]. The former attempts to search for separate characters before joining them and transcribing the complete sequence [31], while the latter focuses on matching the different shapes of the text, correcting for orientation and size differences before transcription.

Luo et al. [27] followed a top-down approach by developing a rectification network combined with an attention-based sequence recognition network. The proposed rectification network helps to correct for orientation in distorted images, reducing the impact of irregular text in text recognition. This approach is similar [26], but without the use of a bidirectional decoder to improve transcription accuracy.

Comparing the performance of text recognizers can be difficult due to the differences in testing and training datasets, software and hardware limitations, computational efficiency and the particular focus of each method. Baek et al. [24] detail these issues alongside problems with the datasets, proposing a unified framework to compare these algorithms.

Their study divides recognition into four stages, which are transformation, feature extraction, sequence modelling and prediction. Following these stages, the models obtain the final transcripted string. The authors provide several combinations of techniques and architectures in each stage, studying their performance and obtaining state-of-the-art results. Furthermore, they analyze the most relevant problems observed on the datasets, highlighting low resolution, irregular text, wrong labelling and curved text as the most common ones.

Low-resolution images present additional challenges to the text recognition problem. Single Image Super-Resolution (SISR) techniques can be used to solve the low-resolution problem. SISR is used in a wide variety of fields, most notably surveillance with identification purposes as well as medical imaging. The resulting high-quality images can be used as input for text recognition algorithms, improving transcription results.

The ICDAR 2015 Competition on Text Image Super-Resolution [32] reported an improvement of over 15% when using Optical Character Recognition on super-resolution enhanced images. The highest scoring method, Super-Resolution Convolutional Neural Network (SRCNN) [33], was based on a deep CNN approach. It extracts batches from the original image after applying bi-cubic interpolation and represents them as feature maps that are matched into each other, representing the high-resolution batch. Following this approach, super-resolution methods have focused on improving performance by increasing network depth with more convolutional layers or reducing the architecture complexity for real-time applications [34].

However, these approaches often miss high-frequency image details, which reduces their performance. More recent works have focused on obtaining a more accurate match between the original image and the super-resolution variant, by implementing sub-pixel convolutional layers or using residual learning [35]. The SRGAN method [36] proposes the use of Generative Adversarial Networks (GAN), implemented with a deep residual network alongside skip-connection. The resulting work generates photo-realistic images of up to four-times scaling.

## 3. Methodology

### 3.1. State-of-the-Art Datasets

To test both the rectification and super-resolution approaches, we used four state-of-the-art datasets commonly referenced by the most recent text recognizers. The SVT dataset [37] was selected due to the presence of low-resolution and blurry images, taken from Google’s Street View and containing a total of 647 text regions. Each image has a 50-word lexicon associated with it, which allows for the improvement of recognition results through string-comparison techniques when the output sequence is not identical to the ground-truth.

For the representation of irregular, curved text, we selected the International Conference on Document Analysis and Recognition (ICDAR) 2015 competition [38] dataset, which contains a total of 2096 images obtained from using Google Glasses in natural environments, containing several noisy and oriented texts. Over 200 of these images also contain irregular text [27]. No lexicon is associated with these images.

In addition to this ICDAR dataset, we also chose the 2013 ICDAR dataset [39], which holds over 1093 crops from scene images with no lexicon associated. We did not perform any filtering on this dataset [27]. Lastly, we added the IIIT5K-Words [40] dataset, which holds 3000 cropped regions from both scene text and born-digital images. Each image has two different lexicons associated, containing 50 and 1000 words, respectively.

**Figure 3:**
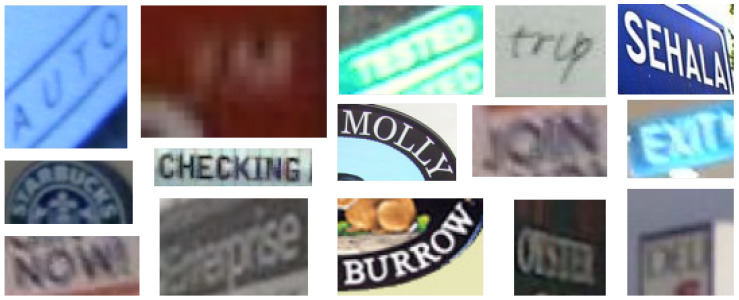
Resolution and orientation issues in state-of-the-art datasets.

Figure 3 illustrates some of the orientation and resolution-based problems that can be found in images taken from these datasets.

### 3.2. Toico-1k

**Figure 4:**
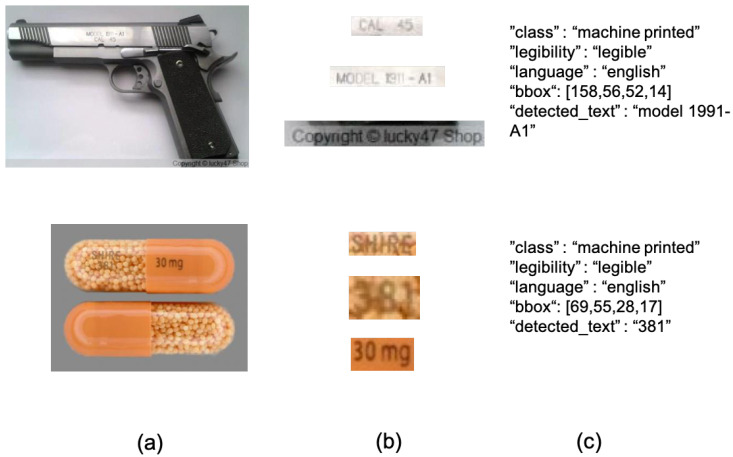
TOICO-1K sample images. (**a**) represents the original image, (**b**) cropped text regions and (**c**) details labelling examples.

To demonstrate the effectiveness of our approach in low-resolution and oriented images, we used our custom dataset [22], named in this paper as TOICO-1K, created specifically for the tasks of text detection and text recognition on images crawled from Tor darknet and which is publicly available in our group website (http://gvis.unileon.es/dataset/tor_images_in_context-1k/). Figure 4 gives examples of the images and the labelling process.

The data creation process was semi-automatic. We took 101 images from the TOIC dataset [41], and generated the first version of TOICO-1K using a text spotting approach, separating both detection and recognition stages. We used the former to assist the task of text region labelling and generation of most of the text bounding boxes, and the latter to obtain an initial transcription of the text detected. Then, we manually inspected the 101 images, and we (i) added missing text regions not automatically detected, (ii) corrected the predictions made by the text recognizer algorithm and (iii) added additional information per text region inspired by [38,39].

We exported all the information to a JSON file, which contains: the type of text found (handwritten or machine-based), whether or not the text is legible, language, number of text regions per image, image dimensions and bounding box locations. The resulting dataset consists of 1101 documented text regions. For our experimentation, we only chose the cropped areas labelled as “legible”, reducing the quantity of cropped regions to 675.

### 3.3. Csa-Text Dataset

The rapid increase in the use of mobile devices and social media brought about an increase in the distribution of CSAM, especially in the darknet. Efficient forensic tools are required for the criminal investigation of such multimedia content and the European funded project Forensic Against Sexual Exploitation of Children (4NSEEK), to which this research work belongs to, is concerned with this problem.

We tested our approach on text retrieved from CSA images that have become highly prevalent worldwide. Many of the CSA images also contain watermark-based text as well as machine printed text that can help identify specific brands, publishers or names. However, they can also contain more obscure texts, which can be hard to retrieve.

**Figure 5:**
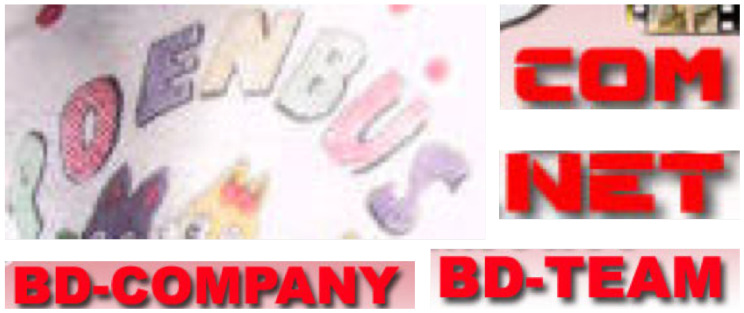
Child Sexual Abuse (CSA)-text dataset sample images.

We created this dataset manually by crawling a total of 232 CSA based images, manually transcribing the regions that contained text and cropping the relevant areas before detailing the text found inside. The resulting dataset has a total of 648 text crops that contain low-resolution images and oriented text. Figure 5 shows examples of text found within this dataset.

### 3.4. Recognition Methods

We selected eight methods to establish an initial text recognition baseline. We chose these methods due to their state-of-the-art results, their focus on computational efficiency [30], their approaches on oriented text, and their implementation of rectification techniques [24,26,27].

Fast Oriented Text Spotting (FOTS) [30] presents an end-to-end text spotting system trained on synthetic images that attempts to integrate both detection and recognition as simultaneous tasks that share information between each other while reducing computational time. The recognition branch is composed of a custom VGG sequence, a bi-directional Long Short Term Memory (LSTM) network and a CTC decoder to obtain the final sequence. This approach allows for a real-time text recognition speed that achieved state-of-the-art results on the ICDAR 2013 and 2015 datasets.

ASTER (Attentional Scene TExt Recognizer with flexible rectification) [26] and MORAN (Multi-Object Rectified Attention Network) [27] focus on the problem of irregular text, which is common in natural scenes and complicates the transcription task. These works resolve this problem through the use of rectification networks.

This technique is relevant in the context of both Tor and CSA based images, due to the common presence of multiple oriented items in the same picture used to express quantity. This approach can also be used to enhance text detectors, correcting the area obtained from the detection and giving feedback to this task, improving the performance of end-to-end systems.

The ASTER recognizer is composed of two stages, namely the initial rectification and the recognition network. When an image is fed to the algorithm, it is rectified using Thin-Plate-Spline (TPS) transformation [42], correcting oriented and perspective text. After the rectified image is obtained, the recognition network processes it and outputs a character sequence. This output is generated using a bidirectional decoder in order to improve transcription accuracy, choosing as the final result the sequence with the highest recognition score.

The MORAN approach is composed of two different networks, the multi-object rectification network (MORN), which rectifies images, and the attention-based sequence recognition network (ASRN).

The rectification network is trained using weak-supervision in order to rectify images with greater distortions. The recognition stage is trained using a custom approach that learns neighbouring features in training, increasing the robustness of the rectification by taking features from both foreground and background context. Finally, both networks are then optimized end-to-end.

Out of the models provided by [24], we tested 5 of their pre-trained configurations, of which 2 used the TPS transformation while 3 did not. In the later stages of the pipeline, the methods alternate using VGG [43] and ResNet [44] as the main feature-extracting neural networks. To improve the extracted features, they enable the use of Bidirectional LSTM, at the cost of computational time.

Lastly, for the prediction stage, they use Connectionist Temporal Classification (CTC) and attention-based prediction, although CTC is more common in the proposed combinations. This approach predicts a character per column of features extracted, modifying blank and repeated characters before producing the final transcription.

### 3.5. Super-Resolution Approaches

Since low-resolution images are prevalent in the forensic field, we selected three super-resolution approaches to try to improve the performance in recognition: (i) Residual Dense Networks (RDN) [35], (ii) Deep CNN with Skip Connection (DCSCN) [34] and (iii) Neural Enhance (NE).

We chose RDN for its focus on exploiting the hierarchical features from all convolutional layers, which is valuable on images with differently scaled objects and aspect ratios. We chose DCSCN for its focus on building a smaller architecture that can lower the computational cost. Finally, we selected NE for its four different models of enhancing images.

Residual Dense Networks [35] propose the use of hierarchical features using residual dense blocks. This structure is made of densely connected layers combined with local feature fusion and local residual learning. The result is a contiguous memory mechanism by acquiring the state of preceding blocks to each layer of the current one. After extracting these local features, dense feature fusion is used to process hierarchical global features before upscaling the final. By combining both local and global features, the proposed structure obtains the final, high-resolution image after upscaling.

The Deep CNN with the Skip Connection algorithm [34] proposes a fully CNN approach in order to decrease power consumption and processing time, focusing on a smaller model with faster and more efficient computation that is suitable for less powerful systems. It is comprised of both a feature extraction network and a reconstruction network.

The extraction network obtains both local and global features using a cascade of CNNs combined with Skip connection layers, with a decreased number when compared to other similar approaches. After joining all of the features, DCSCN uses parallelized CNNs in order to reduce the input dimension before creating the enhanced image.

Lastly, the open-source approach called Neural Enhance (https://github.com/alexjc/neural-enhance) is based on multiple super-resolution techniques attempting to combine all of them in a single implementation. This approach also allows for three different enhance modes (repair, deblur and default), where the default mode allows the possibility for ×2 and ×4 scaling to obtain higher quality images. Combining all of the chosen super-resolution methods, we obtain a total of six possible configurations for resolution enhancement.

## 4. Experimental Results and Discussion

### 4.1. Experimental Setup

We evaluated all the methods on an Intel Xeon E5 v3 computer with 128GB of RAM using an NVIDIA Titan Xp GPU. All methods were implemented under Python3. We ran all of the algorithms using their default configurations. For ASTER and MORAN, we disabled the use of the rectification networks. We did not use any lexicons or dictionaries in our experiments.

We measured the performance of each method according to the percentage of Correctly Recognized Words (CRW). We considered a transcription correct only if both the documented label and the algorithm output are identical. In order to determine the best performing algorithms, we used the Levenshtein distance [45] to measure the total and the standard deviation of the edit distance. The lower the distance, the closer transcribed words are to the documented labels.

After obtaining the initial baseline and identifying the best methods, we separated the images that were not correctly recognized and enhanced them with the use of the super-resolution algorithms. For the NE method, we used each of the repair, deblur and default configurations separately.

**Figure 6:**
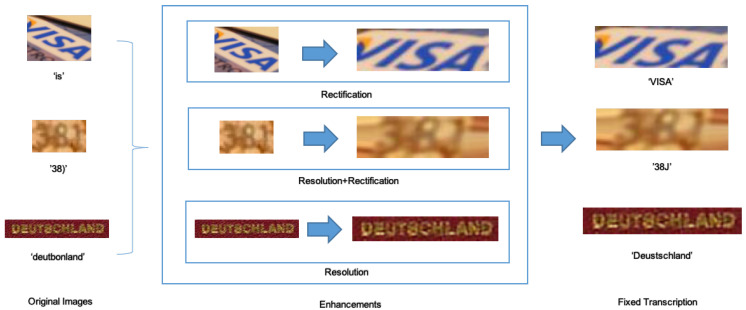
Proposed methodology. Images that were not correctly recognized are enhanced by super-resolution and rectification techniques standalone and in combination.

Lastly, we took the resulting enhanced images and fed them into the ASTER and MORAN recognizers, both with the rectification enabled and disabled in order to obtain the final transcriptions, studying how the proposed techniques enhanced performance when combined or applied separately. A visual representation of our pipeline is shown in Figure 6.

### 4.2. Results and Discussion

The initial recognition results on each of the six datasets are summarized in Table 1. Of the rectification based algorithms, ASTER obtained the best CRW results in all datasets except for IIIT5K, for which MORAN achieved a higher score of 0.9243.

Among the methods proposed by [24], the TPS + ResNet + BiLSTM + ATTN combination obtained the best performance on our TOICO-1K dataset. It also outperformed the MORAN approach on the CSA-text, SVT and IC15 datasets. The pre-trained models that included the TPS transformation achieved higher performance than those which did not. This result validates our rectification-based approach to enhancing text recognition.

Of the methods that did not implement TPS, the Resnet + None + CTC configuration obtained the best performance on all but the IC13 dataset. The use of the ResNet architecture increased results significantly when compared to the VGG approach, even when combined with the BiLSTM technique.

Table 2 presents the total edit distance of the words that were not correctly recognized. This measurement helps indicate how close the methods were on their failure cases, which can be helpful when comparing methods with similar scores.

Using these values, we were able to make a distinction in the methods proposed by [24], identifying the Resnet + None + CTC as the second-best recognizer for our CSA-text dataset since it achieves the least total edit distance. This result implies that the proposed images may not benefit much from the application of rectification techniques.

In both of our custom datasets, FOTS achieved the lowest total edit distance. When compared to the rest of the methods in our CSA-text datasets, this approach greatly reduced the standard deviation of the edit distance. This result suggests that the use of dictionaries alongside FOTS may be desirable to enhance the performance further.

However, when applied on the state-of-the-art datasets, FOTS obtained the highest edit distance, with noticeable increases on the IC15 and IIIT5K datasets. Combined with the large difference in the original recognition scores, ASTER and MORAN remain better approaches despite the reduced word closeness in our datasets.

Table 3 and Table 4 introduce the results of applying the rectification and recognition networks on ASTER and MORAN, respectively. The ASTER recognizer obtained the highest results with rectification enabled on the CSA-text and IIIT5K datasets when combined with NE Repair, SVT and TOICO-1K when using the DCSCN models, and the ICDAR datasets with RDN.

Using ASTER, we obtained a maximum performance score of 0.3170 on TOICO-1K, using the DSCSN approach with rectification, while the CRW score on our CSA-text dataset achieved a 0.6960 using the NE Repair model, which did not improve with the addition of rectification. This result suggests that the images present in our dataset may benefit more from image enhancements rather than orientation correcting techniques.

While the combinations proposed for the MORAN recognizer helped to improve the performance, they only ever outperform ASTER’s original scores in the IC15 dataset using the enhancements provided by the Neural Enhance default ×2 and the RDN approaches with rectification enabled, which obtained a score of 0.7255 over ASTER’s original 0.7235.

Similarly, despite the application of these enhancements, ASTER never surpassed the MORAN recognizer on the IIIT5K dataset. Thus, the application of these enhancements on the recognizers only helped surpass ASTER’s original score by a very small margin.

The MORAN obtained scores of 0.2933 and 0.5448 on our custom TOICO-1K and CSA-text datasets, using the DSCSN model with rectification enabled and the Neural Enhance Default respectively. Unlike ASTER, in our CSA-text dataset combining both techniques either did not improve the performance or decreased it in all the proposed combinations.

Despite this, the MORAN’s rectification network obtained the largest improvement on our chosen datasets when combining both tasks. The RDN and NE Deblur obtained the best results on the IIIT5K and SVT datasets, respectively. On the ICDAR datasets, DCSCN obtained the highest score, tied with the RDN and NE Default approaches for the IC15 and IC13, respectively.

Although the recognition rate increased in all datasets when combining these techniques with the ASTER recognizer, there were cases when the combination of rectification and resolution tasks did not obtain higher results than standalone uses of these techniques. On our TOICO-1K dataset, we obtained a 0.3052 score when applying rectification and 0.2933 using the NE Repair approach. However, when combining both approaches, we obtained a score of 0.3007, which is lower than the single application of rectification.

Using ASTER on TOICO-1K, we obtained lower scores than the simple application of rectification over the combination of both tasks in the NE Repair and Deblur approaches, while for our CSA-text dataset this only affected the RDN approach. However, we observe that with the MORAN recognizer, this behaviour also extended to the state-of-the-art image sets, as can be seen in the IC15, IC13 and IIIT5K datasets.

This performance block could be explained by the alterations of the image carried out by the resolution techniques. When applied to similar characters, these approaches may highlight these alterations by deblurring or enhancing key areas, which can potentially penalize similar character recognition. Such a penalty can be prevented by implementing lexicons, string-matching techniques and the average-edit distance to measure word closeness better and avoid recognition mistakes [23].

Finally, Table 5 and Table 6 show the overall improvements each approach obtains in the given datasets. We obtained an improvement of 3.41% when combining rectification with the DCSCN approach in both the ASTER and MORAN recognizers. For the MORAN method, the same improvement was achieved using RDN and rectification.

Overall, ASTER obtained a larger improvement over MORAN in TOICO-1K, with 2.45% over MORAN’s 2.35%. However, the MORAN obtained a better improvement on our CSA-text dataset, with a 0.93% over ASTER’s 0.77%. The results of combining resolution and rectification in this dataset suggest that resolution is more relevant in these types of images.

On average, ASTER benefits more from the resolution-based approaches, while MORAN improves the performance due to the use of the rectification network. Our experimental results indicate that the ASTER recognizer performs better on the given datasets, except for the IIIT5K, and that both super-resolution and rectification techniques are very close in terms of performance, with the MORAN and ASTER rectification networks obtaining slightly higher results than super-resolution except in images similar to those contained in the CSA-text dataset.

The MORAN obtained lower scores when adding rectification in addition to the resolution, while only the DCSCN method helped improve ASTER’s recognition results in this dataset. The MORAN obtained the highest average improvement on our dataset, with 0.62% over ASTER’s 0.44%.

While none of the approaches decreased the score established over the baseline, when combining the MORAN recognizer and the DCSCN model on the SVT dataset, there was no improvement in the recognition task. This was the only instance in which we did not obtain a higher score when applying the proposed techniques.

On the state-of-the-art datasets, ASTER obtained better improvements than MORAN in all configurations of the SVT dataset, with an average improvement of 1.98% over MORAN’s 0.75%. The highest score in this dataset was obtained using the DCSCN approach combined with rectification.

The RDN approach obtained the best results on the IC15 and IC13 datasets with 3.77% and 2.93%, respectively. The Neural Enhance approach outperformed the other methods on the IIIT5K-Words, with an improvement of 2.07%.

The MORAN obtained the highest score improvement over any dataset on the IC15 dataset with a 4.83% improvement when combined with the RDN and the Neural Enhance approach. On average, the recognizer outperformed ASTER in both the IC15 and IIIT5K datasets with 3.42% and 1.42% CRW improvement.

## 5. Conclusions

In this paper, we address the problem of improving the performance of text recognition for forensic applications assessing the use of rectification networks together with super-resolution techniques. We tested our approach on four state-of-the-art datasets and two custom datasets, a Tor-based image dataset (TOICO-1K), which is publicly available, and a CSAM based dataset, proposing three different environments for text spotting application.

We applied the Levenshtein distance to further differentiate between methods with similar performance. Despite FOTS scoring the lowest total distance in our custom datasets, the performance score difference against the rectification-based approaches is significant enough to ignore word closeness in favour of full-string match performance. However, as shown in our CSA-text dataset, methods could benefit from task-specific dictionaries in order to further enhance their performance.

We studied the combination of different techniques in each stage of the recognition task, as seen in [24]. We identified the combination of the TPS and BiLSTM techniques together with the ResNet CNN and attention-based decoders as the best approach. For images that do not require rectification operations, the addition of BiLSTM to the pipeline could be desirable to enhance recognition performance further. Despite the lower scores achieved by these pipeline combinations, their use is still recommended for real-time systems.

We obtained the maximum improvement on ICDAR 2015 dataset, 4.83%, combining the RDN and the rectification network provided by the MORAN recognizer. In TOICO-1K, DCSCN combined with the rectification improved the baseline results a 3.41% using either of the proposed recognizers. For our CSA-Text dataset, we improved the recognition by 0.93% using only resolution-based techniques.

When analyzed separately, rectification slightly outperforms super-resolution methods on average recognition improvement. Amongst the proposed techniques, Neural Enhance obtained higher results than the rest of resolution techniques when no rectification was involved, with the deblur and default ×2 scaling parameters as the best configurations. However, when combined with the rectification networks, DCSCN obtained the highest improvement scores. These results highlight low-resolution as a relevant challenge for text recognizers, but less relevant than the problem of rotated text.

For our datasets, we conclude that the application of resolution yields better results than rectification on CSAM, due to the higher presence of machine-printed text in low-quality images. In Tor-based images, as objects are presented in multiple orientations to express quantity and product difference, rectification techniques help further enhance the results obtained by text recognizers.

Our results demonstrate that the combination of both tasks improves text recognition in both our real-world problems as well as state-of-the-art datasets, identifying ASTER as the best text recognizer for five out of the six chosen datasets. When combined with super-resolution techniques, ASTER obtained the highest score with a 0.3170 and 0.6960 for TOICO-1K and CSA-text, respectively, outperforming all other approaches.

Based on our results, we recommend the use of rectification techniques over super-resolution approaches in state-of-the-art datasets, as the correction of the images obtained higher overall improvements. However, images similar to those of the IC15 dataset can still benefit from the standalone use of super-resolution techniques, although the enhancements are lower than those of the image correction approach.

For Tor-based images, we recommend the use of the DCSCN method, as it obtained higher average scores when used standalone with the ASTER recognizer, as well as when combined with the rectification networks. In the case of CSA images, resolution techniques should be prioritized over the orientation approach.

Our future work will focus on the study of text recognition based problems, such as aspect ratio and partial occlusion. The evaluation process is also a relevant problem, which can penalize models that do not fully match words with similar characters, such as the ‘o’ letter and the number zero, which are common in both TOICO-1K and CSA-text images and the difficult recognition of keywords linked to illegal activities. After transcription, NLP techniques can be used to process and classify text, which can be useful to identify sellers and entity names in both the CSA and Tor environments.

Lastly, the labelling of the datasets can also pose a performance decrease for text recognizers, as multiple state-of-the-art datasets [24] have been reported of including various mistakes on their documented labels.

## Figures and Tables

**Table 1:** Text recognition performance result, using the Correctly Recognized Words (CRW) measurement. The best score on each dataset is highlighted in bold.

Method	TOICO-1K	CSA-Text	SVT	IC15	IC13	IIIT5K
FOTS	0.2074	0.3889	0.5255	0.2499	0.7292	0.5700
MORAN	0.2652	0.5355	0.8671	0.6771	0.8573	**0.9243**
ASTER	**0.2830**	**0.6883**	**0.8825**	**0.7235**	**0.8829**	0.8413
None + ResNet + None + CTC	0.2163	0.5448	0.8377	0.6283	0.8280	0.8397
None + VGG + BiLSTM + CTC	0.2089	0.5417	0.8207	0.6119	0.8289	0.8273
None + VGG + None + CTC	0.1319	0.3858	0.7558	0.5060	0.7749	0.7600
TPS + ResNet + BiLSTM + ATTN	0.2637	0.5417	0.8702	0.6984	0.8545	0.8740
TPS + ResNet + BiLSTM + CTC	0.2385	0.5448	0.8624	0.6733	0.8417	0.8618

**Table 2:** Total edit distance and standard deviation results per method on each dataset.

Method	TOICO-1K		CSA-Text		SVT		IC15		IC13		IIT5K	
	ED	Std	ED	Std	ED	Std	ED	Std	ED	Std	ED	Std
FOTS	2859	5.039	957	1.635	846	2.109	5346	2.221	631	1.701	2918	1.709
MORAN	3197	5.367	1783	4.112	203	2.034	1716	2.026	277	1.200	515	2.816
ASTER	2914	5.496	2530	5.405	165	2.003	1522	1.971	248	1.356	774	1.821
None + ResNet + None + CTC	3173	5.595	1386	2.678	190	1.488	1752	1.731	310	1.212	836	1.725
None + VGG + BiLSTM + CTC	3608	6.590	1413	2.864	235	1.618	1934	1.827	314	1.229	978	2.083
None + VGG + None + CTC	3547	5.557	2059	4.446	330	1.598	2439	1.703	421	1.182	1376	1.851
TPS + ResNet + BiLSTM + ATTN	3219	5.832	1568	2.991	169	1.485	1579	2.051	283	1.578	757	2.529
TPS + ResNet + BiLSTM + CTC	3044	5.343	1553	2.975	158	1.396	1567	1.874	287	1.323	701	1.808

**Table 3:** CRW metric combining ASTER with super-resolution. Residual Dense Networks (RDN), Deep CNN with Skip Connection (DCSCN) and (iii) Neural Enhance (NE) combinations are applied over the baseline. The best score on each dataset is highlighted in bold.

Method	TOICO-1K	CSA-Text	SVT	IC15	IC13	IIIT5K
ASTER (Baseline)	0.2830	0.6883	0.8825	0.7235	0.8829	0.8413
ASTER (Baseline) + Rectification	0.3052	0.6914	0.9042	0.7424	0.8984	0.8540
RDN	0.2993	0.6898	0.8934	0.7453	0.8957	0.8483
RDN + Rectification	0.3096	0.6898	0.9042	**0.7612**	**0.9122**	0.8557
DCSCN	0.3037	0.6898	0.8995	0.7414	0.8930	0.8470
DCSCN + Rectification	**0.3170**	0.6944	0.9104	0.7574	0.9076	0.8560
NE Repair	0.2933	**0.6960**	0.8964	0.7351	0.8939	0.8543
NE Repair + Rectification	0.3007	**0.6960**	0.9042	0.7487	0.9021	**0.8620**
NE Deblur	0.2933	0.6914	0.9011	0.7380	0.8911	0.8540
NE Deblur + Rectification	0.3007	0.6914	0.9057	0.7477	0.9039	0.8613
NE Default ×2	0.2993	0.6898	0.8964	0.7453	0.8939	0.8477
NE Default ×2 + Rectification	0.3126	0.6914	0.9042	0.7598	0.9058	0.8580
NE Default ×4	0.3022	0.6898	0.8949	0.7438	0.8893	0.8493
NE Default ×4 + Rectification	0.3096	0.6914	0.9042	0.7593	0.9039	0.8590

**Table 4:** CRW metric combining MORAN with super-resolution. The best score on each dataset is highlighted in bold.

Method	TOICO-1K	CSA-text	SVT	IC15	IC13	IIIT5K
MORAN (Baseline)	0.2652	0.5355	0.867	0.6771	0.8573	0.9243
MORAN (Baseline) + Rectification	0.2919	0.5370	0.8733	0.7173	0.8774	0.9430
RDN	0.2785	0.5417	0.8717	0.7018	0.8664	0.9323
RDN + Rectification	**0.2993**	0.5417	0.8764	**0.7255**	0.8756	**0.9450**
DCSCN	0.2785	0.5417	0.8671	0.6936	0.8628	0.9297
DCSCN + Rectification	**0.2993**	0.5370	0.8764	0.7206	**0.8792**	0.9443
NE Repair	0.2711	0.5432	0.8733	0.6984	0.8646	0.9327
NE Repair + Rectification	0.2800	0.5432	0.8794	0.7158	0.8747	0.9430
NE Deblur	0.2726	0.5401	0.8748	0.6999	0.8673	0.9347
NE Deblur + Rectification	0.2800	0.5386	**0.8810**	0.7115	0.8728	0.9417
NE Default ×2	0.2770	**0.5448**	0.8702	0.7013	0.8692	0.9343
NE Default ×2 + Rectification	0.2889	0.5432	0.8748	**0.7255**	**0.8792**	0.9447
NE Default ×4	0.2830	0.5432	0.8794	0.6989	0.8701	0.9353
NE Default ×4 + Rectification	0.2933	0.5432	0.8733	0.7250	0.8756	0.9443

**Table 5:** Text recognition improvement on ASTER, measured as the difference between the best-case result and the original baseline. Avg presents the average improvement per dataset (columns) and method (rows).

Approach	TOICO-1K	CSA-Text	SVT	IC15	IC13	IIIT5K	Avg
Rectification	2.22%	0.31%	2.16%	1.88%	1.56%	1.27%	1.57%
RDN	1.63%	0.15%	1.08%	2.17%	1.28%	0.70%	1.17%
RDN + Rectification	2.67%	0.15%	2.16%	3.77%	2.93%	1.43%	2.19%
DCSCN	2.07%	0.15%	1.70%	1.79%	1.01%	0.57%	1.22%
DCSCN + Rectification	3.41%	0.62%	2.78%	3.38%	2.47%	1.47%	2.36%
NE	1.93%	0.77%	1.85%	2.17%	1.10%	1.30%	1.52%
NE + Rectification	2.96%	0.77%	2.32%	3.62%	2.29%	2.07%	2.34%
Average Improvement	2.45%	0.44%	1.98%	2.82%	1.85%	1.26%	/

**Table 6:** Text recognition score improvements for the MORAN recognizer.

Approach	TOICO-1K	CSA-Text	SVT	IC15	IC13	IIIT5K	Avg
Rectification	2.67%	0.16%	0.62%	4.01%	2.01%	1.87%	1.89%
RDN	1.33%	0.62%	0.46%	2.46%	0.91%	0.80%	1.10%
RDN + Rectification	3.41%	0.62%	0.93%	4.83%	1.83%	2.07%	2.28%
DCSCN	1.33%	0.62%	0.00%	1.64%	0.55%	0.53%	0.78%
DCSCN + Rectification	3.41%	0.15%	0.93%	4.35%	2.20%	2.00%	2.17%
NE	1.78%	0.93%	0.77%	2.42%	1.28%	1.10%	1.38%
NE + Rectification	2.81%	0.77%	1.39%	4.83%	2.20%	2.03%	2.34%
Average Improvement	2.35%	0.62%	0.75%	3.42%	1.50%	1.42%	/
